# The Profession of Medicine: Its Study, and Pratice; Its Duties, and Rewards

**Published:** 1897-06

**Authors:** 


### Reviews of Books.

**The Profession of Medicine: Its Study, and Practice; its Duties, and Rewards.** By CHARLES WEST, M.D. Pp. x., 115. London: Kegan Paul, Trench, Trübner, & Co., Ltd. 1896.

Dr. West's high medical record, and his official connection with the College of Physicians, render his views on the study and practice of medicine peculiarly valuable. He looks back to the advantages of apprenticeship in the early training of a medical man, and also strongly advises some knowledge of the principles of the collateral sciences. His advice as to work in the wards, note-taking, and the importance of good manners, even towards the most debased patients in hospital, shows that he wishes students to be not only good doctors, but gentlemen in the highest sense. The greater part of his book is devoted to the practice of medicine. His views on the necessity of long-suffering and of quick observation, on a hopeful manner to the patient and his friends, with suggestions as to consultations, note-keeping, and professional secrecy, are wise and eminently sensible, if strict. As regards professional secrecy, he clings to the great dictum of the University of Paris in 1602, "*Ægrorum arcana, visa, audita, intellecta, eliminet nemo.*" Dr. West's exceptions would be, where silence would condemn an innocent person, or would fail to detect crime. He touches with a masterly hand on some of the difficulties of the day, consultation with homœopaths, provident dispensaries, fees, hospital abuse, and friendly societies. The whole tenor of the book is to show that so-called medical etiquette is common honesty and fair dealing to one another; and the injunctions come well from a former senior censor of the College of Physicians, who was long revered by a large circle of his professional brethren.

